# Dual-layer spectral CT for iodine mapping and surrogate perfusion evaluation in cerebrovascular disease

**DOI:** 10.1007/s00330-025-11858-w

**Published:** 2025-07-16

**Authors:** Niclas Schmitt

**Affiliations:** Department of Neuroradiology, Heidelberg University Hospital, Heidelberg, Germany

## Background and clinical relevance

Cerebral perfusion imaging plays a critical role in managing cerebrovascular diseases, including ischemic stroke, transient ischemic attack, and chronic cerebrovascular disorders [1, 2]. The primary objective of cerebral perfusion computed tomography (CTP) is to assess essential parameters such as cerebral blood flow (CBF), cerebral blood volume (CBV), and mean transit time (MTT). These parameters are invaluable in identifying ischemic regions, determining the extent of damage, and guiding therapeutic interventions [3]. However, while conventional CTP (cCTP) provides valuable diagnostic information, it is associated with high radiation doses, which raises concerns for patient safety, particularly for those requiring repeated imaging [4].

The introduction of dual-layer spectral computed tomography (DLCT) enables high-quality spectral imaging, including the generation of iodine density (ID) maps and virtual monochromatic images (VMIs). ID maps may serve as a static surrogate of tissue perfusion but lack time-resolved parameters such as CBF or MTT. [5]. While DLCT has shown promise in improving tissue differentiation and providing more detailed imaging, studies examining the relationship between DLCT and cerebral CTP are limited [6]. The potential of DLCT to deliver quantitative data, such as ID maps, may complement existing techniques and, in selected cases, reduce or even obviate the need for cCTP by offering contrast-based information relevant to cerebrovascular assessment. Moreover, this approach may reduce radiation exposure, whereas the combined use of both techniques results in higher cumulative radiation doses.

In this context, the study by Liu et al marks a step in assessing the feasibility of DLCT-derived perfusion parameters as a reliable alternative to cCTP [7]. By comparing the consistency of perfusion maps from cCTP and spectral CTP (sCTP) derived from the same high-dose dynamic dataset using VMI reconstructions and evaluating the correlation between perfusion parameters and ID from DLCT angiography, Liu et al offer valuable insights into DLCT’s potential to assess cerebral perfusion and iodine distribution in patients with cerebrovascular disorders.

## Summary of the findings

Liu et al retrospectively analyzed 163 patients with cerebrovascular disorders, comparing perfusion parameters from cCTP and sCTP derived from the same dynamic dataset, and relating them to ID maps from CTA. The results revealed several key findings:

1. **Potential for radiation dose reduction:** A reduction of 39% in radiation exposure was observed for the spectral data obtained from single-phase CTA used for iodine mapping in contrast to cCTP. This might be especially beneficial in scenarios requiring repeated imaging, such as chronic cerebrovascular monitoring or stroke follow-up.
2. **Consistency of perfusion parameters:** Despite differences in reconstruction technique, good agreement was found between sCTP and cCTP parameters. Relative CBF, relative CBV, and other perfusion values showed strong correlations, particularly at 60 keV VMIs. These findings suggest that DLCT-based VMI reconstructions can reliably estimate perfusion metrics derived from dynamic acquisitions.
3. **Clinical relevance:** ID maps from DLCT may serve as static surrogates for cCTP imaging, although they do not provide dynamic parameters such as CBF or MTT. This finding might be helpful in patients for whom CTP is contraindicated or impractical.

## Clinical implications and future perspectives

The findings may have important clinical implications for balancing radiation exposure with diagnostic accuracy in cerebrovascular imaging. The ability to perform low-dose imaging without compromising quality is crucial, especially for patients needing serial assessments.

Earlier studies have shown that VMIs can improve brain tissue depiction, enhance vascular contrast, and reduce artifacts [8]. In the context of spectral CT, VMIs derived from perfusion datasets may improve contrast resolution and image quality. Although their diagnostic utility in cerebral perfusion remains to be established, the data presented by Liu et al support their role as a valuable adjunct to conventional imaging, without replacing dynamic perfusion techniques.

An advantage of spectral CT is its ability to assess both perfusion and vascular status in one scan [5]. Acquiring iodine-based surrogate data and vascular information from a single CTA may streamline clinical workflows and reduce the need for multiple studies, particularly relevant in acute settings where time and dose are critical factors.

Previous studies demonstrated the feasibility of DLCT-based perfusion imaging in other organs. For example, Stiller et al demonstrated that iodine maps correlate well with pancreatic perfusion, and Pelgrim et al showed that myocardial iodine concentration can serve as a semi-quantitative perfusion marker [9, 10]. These findings support the use of iodine quantification as a surrogate for dynamic perfusion. However, brain-specific applications have remained underexplored. This study fills that gap by evaluating DLCT-derived ID maps for cerebral perfusion, expanding the use of this technique into neuroimaging.

A single CTA scan generating ID maps may offer supportive insight into cerebral iodine distribution, particularly in patients for whom CTP is contraindicated or unavailable. However, these static maps do not replace time-resolved perfusion parameters such as CBF or MTT, which remain essential for stroke triage [10, 11].

Although DLCT-derived ID shows good consistency with CTP, its role as a true surrogate for CBF requires prospective, multicenter validation. The current study included patients with varying diseases and uncertain perfusion status, making it difficult to fully assess diagnostic performance. Future research should focus on well-defined cerebrovascular conditions and disease stages to better understand the clinical value of DLCT iodine maps. Optimizing VMI settings and defining robust thresholds for relative ID will also be critical for broader implementation and accurate outcome prediction.

## Conclusion

Liu et al demonstrate that spectral VMI-based perfusion parameters correlate well with cCTP when derived from the same high-dose dataset. ID maps from single-phase DLCT CTA may serve as a low-dose adjunct or surrogate to cCTP, although they provide only static contrast information and are not a replacement for dynamic perfusion imaging. The ability to extract both iodine and spectral data from a single CTA scan offers the potential for additional functional insight in selected cases. While DLCT offers promising options for workflow streamlining and radiation sparing in selected scenarios, its diagnostic value requires further validation. In particular, prospective multicenter studies with standardized acquisition protocols, defined diagnostic thresholds, and more homogeneous patient cohorts are essential to determine the clinical utility of DLCT-derived ID maps as an alternative to cCTP in selected cases.
